# Emerging Fungal Infections of the Central Nervous System in the Past Decade: A Literature Review

**DOI:** 10.3390/idr16050076

**Published:** 2024-10-09

**Authors:** Rita Lino, André Rodrigues Guimarães, Estela Sousa, Mariana Azevedo, Lurdes Santos

**Affiliations:** 1 Infectious Diseases Department, Hospital de São João—Unidade Local de Saúde São João, 4200-319 Porto, Portugal; andrerodriguesguimaraes@gmail.com (A.R.G.); estelacarvalhosou63@gmail.com (E.S.); mariana_campos_azevedo@hotmail.com (M.A.); maria.lurdes.uci@gmail.com (L.S.); 2 Faculty of Medicine, University of Porto, 4200-319 Porto, Portugal; 3 ESCMID Study Group for Infectious Diseases of the Brain (ESGIB), 4051 Basel, Switzerland; 4 ESCMID Study Group for Infections in Compromised Hosts (ESGICH), 4051 Basel, Switzerland

**Keywords:** fungi, central nervous system, *Blastomyces*, *Candida auris*, *Lomentospora*, *Scedosporium*, *Sporothrix*, *Talaromyces*, *Trichosporon*

## Abstract

Introduction: Invasive fungal infections affecting the central nervous system (CNS) are a major health concern worldwide associated with high mortality rates. Their increased incidence is largely due to an increase in the vulnerable immunocompromised population, changing environmental factors, and development of more accurate diagnostic methods. The aim of this article is to identify fungal causes of CNS infections that are recently emerging or have the potential to become emerging pathogens in the near future, as well as their clinical characteristics, including: *Candida auris*, *Trichosporon* spp., *Blastomyces* spp., *Sporothrix* spp., *Talaromyces marneffei*, *Lomentospora prolificans*, and *Scedosporium* spp. Methods: A review of the literature in PubMed in the last ten years was conducted to identify central nervous system infections caused by each of these fungi. Results: The review identified 10 cases caused by *C. auris*, 5 cases by *Trichosporon* spp., 82 cases by *Blastomyces* spp., 36 cases by *Sporothrix* spp., 21 cases by *T. marneffei*, 22 cases by *Lomentospora prolificans,* and 42 cases by *Scedosporium* spp. Discussion: The exact burden of these diseases remains difficult to ascertain, but their apparent rise underscores the urgent need for improved diagnostic, treatment, and management strategies against CNS fungal pathogens to improve outcomes against these life-threatening infections.

## 1. Introduction

Invasive fungal infections have shown increasing incidence, emerging as a growing cause of concern worldwide. In 2022, the World Health Organization developed the first fungal priority pathogens list in order to encourage global awareness and response to fungal infections [1].

The development of sophisticated genetic and molecular methods has enhanced our knowledge of fungal pathogens and their diversity [2], but a lack of accessible and high-quality diagnostic methods still hampers our ability to accurately diagnose and estimate the true burden of these diseases.

Several other factors have contributed to the increased incidence of fungal infections, with a significant contributor being the rise in the number of immunocompromised individuals. Advances in medical therapies for people living with cancer, autoimmune diseases, HIV, solid or stem cell transplants, or invasive devices have extended and improved life expectancy in these populations. However, these advancements have also led to higher susceptibility to severe fungal infections among these individuals [3].

Environmental determinants such as climate change, migration, and extensive antimicrobial use in agriculture and medicine also play a role. These factors have contributed to shifts in the geographical distribution of fungal diseases and the emergence of drug-resistant pathogens [4].

Fungi are being increasingly recognized as causes of central nervous system (CNS) infections. Their propensity to affect the most vulnerable patients, diagnostic challenges, and limited therapeutic options contribute to high mortality rates. The role of the more common causes of invasive fungal infections, such as *Cryptococcus* spp., *Aspergillus* spp., *Mucorales*, *Histoplasma* spp., and most species of *Candida*, has been well-known and extensively studied through the years.

This article aims to provide an overview of fungal pathogens capable of causing CNS infection that are recently emerging and/or have the potential to become emerging pathogens in the near future, including: *Blastomyces* spp., *Candida auris*, *Lomentospora prolificans*, *Scedosporium* spp., *Sporothrix* spp., *Talaromyces marneffei,* and *Trichosporon* spp.

## 2. Materials and Methods

A narrative literature review was conducted to explore the clinical characteristics and reported cases of emerging fungal pathogens causing CNS infections in the last decade. Articles were screened based on the following criteria: increased incidence, expanding or shifting geographical distribution, and evidence of multiple reports of CNS involvement. Fungi previously well-established as important causes of CNS infection, without evidence of disproportionate increase in the number of infections in the last ten years, or that showed no apparent potential to become a major health concern, were excluded. A final selection of seven unconventional fungi with growing clinical relevance in CNS infections was made to be the focus of this article, including *Blastomyces* species, *Candida auris*, *Lomentospora prolificans*, *Scedosporium* species, *Sporothrix* species, *Talaromyces marneffei*, and *Trichosporon* species. A literature search was performed using the MEDLINE database via PubMed to identify articles describing fungal central nervous system infections by these microorganisms published from 2013 to December 2023. Citations for the articles found were reviewed for additional cases. Confirmed, probable, and presumptive cases of CNS infections by these agents were included. Any CNS involvement was included, namely meningitis, encephalitis, stroke syndrome, or space-occupying lesions. Only articles written in English, Portuguese, or Spanish that involved human cases were included. A summary of each fungus’ broad clinical characteristics is presented in Table 1. A review of the literature of the number of CNS infections by these fungi in the last decade is summarized in Table 2 and Table 3.

## 3. Yeasts

### 3.1. Candida auris

#### 3.1.1. Definition and Epidemiology

*Candida auris* is an emerging yeast first described in 2009 from the ear discharge of a patient in Japan [110]. Since then, it has emerged and rapidly spread as a cause of invasive infections and outbreaks in six continents, sparing only Antarctica [4]. Globally, *C. auris* cases and ongoing transmission have been steadily increasing in recent years [111], contributing to the epidemiological change in the landscape of invasive candidiasis. The main cause of invasive candidiasis has classically been *C. albicans,* but there is now a growing prevalence of non-albicans species [4].

The US Centers for Disease Control and Prevention designated *C. auris* an urgent threat due to multiple reasons, namely its high prevalence of multidrug resistance, ability to cause invasive infections with high mortality rates, and easy transmissibility between patients in healthcare settings [112].

Identified risk factors for *C. auris* infection include immunosuppression, multiple or prolonged healthcare contact, the presence of indwelling devices, the use of broad-spectrum antibiotics or antifungal agents, and skin colonization [111,113].

#### 3.1.2. Clinical Features

*C. auris* can colonize patients’ skin and survive in surfaces and devices. It has a wide clinical spectrum, ranging from asymptomatic colonization to disseminated infection. Candidemia is frequent, with other manifestations being urinary tract infections, otitis, wound infections, skin abscesses, osteomyelitis, myocarditis, catheter or device-associated infections, or meningitis [113]. CNS infection is rare and can be either secondary to hematogenous dissemination or associated with neurosurgical procedures [9]. It usually manifests as meningitis, ranging from acute to chronic presentations.

Among case reports, literature reviews and outbreak reports, a total of 10 cases of CNS infection due to *C. auris* were found [5,6,7,8,9,10,11,12]: 6 neurosurgical devices infections, 3 meningitis, and 1 ventriculitis. Nevertheless, considering the increasingly high number of outbreaks reported worldwide, this is probably an underestimation of the true burden the CNS manifestations of *C. auris*.

#### 3.1.3. Diagnosis

The gold standard for diagnosing invasive candidiasis involves obtaining positive cultures from normally sterile sites, which allows for species identification and susceptibility testing. For CNS infections, diagnosis should be based on culture growth from appropriate samples such as cerebrospinal fluid (CSF) or positive histopathology [114]. Polymerase chain reaction (PCR)-based methods have also proven to be sensitive and fast at detecting *C. auris*, which is increasingly useful for both diagnostic and screening purposes [115].

#### 3.1.4. Treatment

*C. auris* consistently demonstrates elevated minimal inhibitory concentrations (MICs) to fluconazole, along with varying susceptibility to other triazoles, echinocandins, and amphotericin B. It is estimated that around 90% of *C. auris* isolates are resistant to fluconazole, 30% to amphotericin B, and up to 5% to echinocandins [111,113]. Some isolates have been found to be resistant to all three major antifungal drugs. Although resistance to echinocandin can be rare in some regions, its prevalence is increasing as well as the prevalence of pan-resistant isolates.

Echinocandins are recommended as the first-line treatment for invasive *C. auris* infection [114]. Antifungal susceptibility testing is essential to further optimize treatment. Acquired resistance, including resistance to echinocandins, can develop during treatment and is a significant concern in these infections [113,116].

However, due to the inadequate penetration of echinocandins across the blood–brain barrier, these antifungal agents are not recommended as the primary treatment for CNS infections, specifically meningitis. In CNS infections due to *C. auris*, treatment should be guided by susceptibility testing. Options may include azoles, amphotericin B, or even flucytosine. There is a lack of evidence and randomized clinical trials to determine the optimal therapy for CNS infections caused by *C. auris*. From the cases reviewed, combination therapy with two or more effective antifungals has often been employed, but outcomes have been inconsistent. Singhal et al. report a case of nosocomial CSF shunt infection due to *C. auris* refractory to systemic echinocandin and flucytosine, which only resolved after the addition of oral voriconazole and intrathecal caspofungin [12].

Novel antifungals like ibrexafungerp and fosmanogepix have demonstrated in vitro and in vivo efficacy against *C. auris*, indicating they may offer promising therapeutic options in the future [113]. Fosmanogepix specifically shows excellent concentrations in the eye and CNS, contrary to ibrexafungerp that doesn’t seem to penetrate the blood–brain barrier.

#### 3.1.5. Prognosis

Invasive disease due to *C. auris* is estimated to have a mortality rate between 30% and 60%, although it is challenging to determine the attributable mortality rate of this disease due to the frequent serious concomitant comorbidities of these patients [115]. A recent multicenter case–control study in New York did not show an increase in 30-day or 90-day mortality rate in bloodstream infections due to *C. auris* in comparison to bloodstream infections caused by other *Candida* species. However, all *C. auris* isolates in this study were echinocandin susceptible, which was the antifungal class used initially in all cases [117].

### 3.2. Trichosporon Species

#### 3.2.1. Definition and Epidemiology

*Trichosporon* species are basidiomycetous yeast-like fungi ubiquitous in nature [118]. These species are predominantly found in tropical and temperate areas and thrive in various substrates, including soil, air, rivers, lakes, seas, decomposing wood, bird droppings, pigeons, bats, scarab beetles, and cattle [118]. These organisms can also be present in various parts of the human body, including the gastrointestinal tract, respiratory tract, skin, and vagina [118].

The genus *Trichosporon* comprises approximately 50 species. However, only a few standout as potential human pathogens, namely *T. asahii*, *T. inkin*, *T. asteroides*, *T. cutaneum*, *T. mucoides*, *T. ovoides*, *T. pullulans,* and *T. loubieri* [119]. Other species rarely cause human disease [13]. The most common species recognized to cause human infections is *T. asahii* [120].

*Trichosporon* species are usually related to superficial infections. Risk factors for acquiring these mycoses are humidity and poor hygiene [120]. Nevertheless, certain species can cause invasive infections, with risk factors including primary or acquired immunodeficiency syndromes, neutropenia and other neutrophil abnormalities, hematologic malignancies, especially acute myeloid leukemia, chemotherapy, high dose corticosteroids, burns, broad spectrum antibiotics, and invasive devices [14]. Owing to the rise in the immunocompromised population, largely due to advancements in medicine, the incidence of invasive disease associated with *Trichosporon* species is also increasing, hence the clinical importance of these fungi as emerging opportunistic agents [119].

Central nervous system invasion was first documented in 1970, presenting as a brain abscess [121], and since then, only a limited number of cases have been reported. In fact, trichosporonosis with central nervous system complications is rare in immunocompromised patients and extremely rare in immunocompetent patients and it seems that its geographical distribution is higher in Asia [15,16].

Five cases of central nervous system infection caused by *Trichosporon* species were reported in the last ten years [13,14,15,16,17]: two cases by *T. asahii* [15,16], one in an immunocompetent host and the other in an immunocompromised host, two cases by *T. inkin* [14,17], both in immunocompromised hosts, and one case of brain abscesses by *T. dohaense* in a diabetic patient treated with systemic corticosteroids for the treatment of COVID-19 [13].

#### 3.2.2. Clinical Features

*Trichosporon* species typically cause superficial infections, including conditions such as white piedra, onychomycosis, and interdigital and inguinocrural lesions [119].

In situations where systemic invasion occurs, typically by hematogenous spread and usually in the setting of primary or secondary immunodeficiency, fungemia is the main presentation. Nonetheless, inflammation and abscess formation in various organs and tissues, such as urinary tract infections, peritonitis, endocarditis and arthritis, can occur [118].

Central nervous system involvement is rare, even within the context of immunosuppression or neurosurgical procedures. However, it is noteworthy that the sole reported case to date of an intraventricular fungal ball by *T. asahii* occurred in an immunocompetent young patient [15]. Brain abscesses, meningitis, and hydrocephalus represent the most frequent manifestations of *Trichosporon* spp. central nervous system infections.

In a study analyzing the nervous tissue of 10 multiple sclerosis (MS) patients and 9 healthy controls, *T. mucoides* was found in the central nervous system of the majority of the MS patients but not in the healthy controls, raising the question of whether disseminated fungal infections may play a role in the etiology of multiple sclerosis, or if MS might predispose to trichosporonosis [122].

#### 3.2.3. Diagnosis

The gold standard for the diagnosis of trichosporonosis involves the cultural growth or the identification by direct microscopy of fungal elements consistent with *Trichosporon* spp. (such as hyphae, pseudohyphae, arthroconidia, and blastoconidia) from blood, cerebrospinal fluid and/or tissue biopsy specimens. However, obtaining biopsy specimens can be challenging in specific cases. Additionally, performing cultures is time-consuming and fails not only to differentiate between colonized and infected patients, but also to identify the specific species within the *Trichosporon* genus [118]. Consequently, molecular methods, including nucleotide sequence-based analysis and protein fingerprint analysis by mass spectrometry, have been increasingly developed attempting to overcome these challenges [118].

In the case of suspected central nervous system infection by *Trichosporon* spp., imaging, ideally a magnetic resonance imaging (MRI), is an essential tool, as it may identify hydrocephalus, circular ring-enhancing brain lesions, abnormal meningeal enhancement, or plaque-like enhancing medullar lesions.

#### 3.2.4. Treatment

Treating patients with trichosporonosis poses a challenge as an optimal treatment has not yet been established. There are limited in vitro and in vivo data on antifungal drugs against clinically relevant *Trichosporon* species. These fungi are notoriously resistant to echinocandins and have reduced susceptibility to flucytosine. Amphotericin B is frequently associated with high MIC values [17]. It is a noteworthy fact that *Trichosporon* spp. can produce biofilm, presenting a further challenge in the treatment of these fungi, as it confers increased resistance to antifungals [15].

Nonetheless, the European Society of Clinical Microbiology and Infectious Diseases and the European Confederation of Medical Mycology joint guidelines for the diagnosis and management of rare invasive yeast infections recommends voriconazole based on limited quality of evidence [123].

#### 3.2.5. Prognosis

Trichosporonosis has a mortality rate between 50% and 80%. The outcome usually depends on the immune status of the host and the extent of infection [124].

## 4. Dimorphic Fungi

### 4.1. Blastomyces Species

#### 4.1.1. Definition and Epidemiology

*Blastomyces* is a genus of thermally dimorphic fungi that grow as a mold in the environment and converts to yeast in the organism. It comprises multiple species with different geographic distributions that usually inhabit soils near waterways. The most common species, by far, is *Blastomyces dermatitidis*. Its clinical aspects have been extensively described for decades, but there have been a growing number of infections in regions previously considered to be non-endemic. *B. dermatidis* is mainly seen in North America in the midwestern, southeastern, and southcentral regions of the United States of America (USA) and midwestern Canadian provinces [125]. However, its geographic range appears to be expanding with new regions at risk in North America, such as the state of New York, Quebec and Saskatchewan [126,127].

Additionally, advances in molecular diagnostic methods have allowed for the recent identification and addition of other species to the genus *Blastomyces* that are microbiologically, clinically, and geographically different from *B. dermatitidis* [2,128]. Recent genetic analysis has been able to identify infections by *B. gilchristii*, *B. percursus*, *B. emzantsi,* and *B. helicus* (formerly *Emmonsia helica*) outside the typical geographic range of *B. dermatidis*, including some that were previously thought to be caused by this last species. The remaining species within this genus have different geographic distributions, as described in Table 1 [43,44,125,129]. The geographic reach and epidemiology of these species are still largely unknown, although they appear to be significantly less prevalent and differ from *B. dermatidis* in certain clinical and histopathological features [129].

Amongst case reports and retrospective studies, 82 cases of CNS blastomycosis were found [18,19,20,21,22,23,24,25,26,27,28,29,30,31,32,33,34,35,36,37,38,39,40,41,42,43,44,45]. Most were either caused by *B. dermatidis*, did not specify or could not identify the species of *Blastomyces.* Regarding infections caused by *Blastomyces* species other than *B. dermatidis*, we found 2 cases of CNS involvement by *B. helicus* and 1 by *B. percursus* [43,44,45].

#### 4.1.2. Clinical Features

The clinical, treatment and diagnostic knowledge of blastomycosis has been predominantly based on infections caused by *B. dermatidis*. Infection typically occurs through the inhalation of conidia, with the lungs being the primary affected organ. Dissemination outside the lungs can occur in approximately 25 to 50% of *B. dermatidis* infections, usually to the skin, bones, or genitourinary system [130]. *B. percursus* are clinically distinct from *B. dermatidis,* as they usually cause a higher proportion of bone and cutaneous manifestations [129]. CNS involvement is reported in approximately 5 to 10% of extrapulmonary cases of blastomycosis caused by *B. dermatidis*. This can occur secondary to hematogenous dissemination or, less frequently, as an isolated manifestation [131]. In contrast, in a review of 10 human cases of infection due to *B. helicus*, CNS involvement was confirmed in 2 of them [44]. However, further evidence is needed to understand if *B. helicus* might have a higher propensity to CNS focalization.

CNS blastomycosis can present as meningitis, meningoencephalitis, ventriculitis, intracranial, epidural, spinal cord mass lesions, or a combination of these conditions. Despite the rarity of CNS involvement, the propensity of *Blastomyces* spp. to disseminate through hematogenous spread establishes it as a potential cause of CNS disease, particularly given its expanding geographical range.

Generally, blastomycosis predominantly affects immunocompetent patients, with the exception of *B. helicus*, which most commonly affects immunocompromised hosts [130,131]. Nevertheless, severe forms of blastomycosis, including CNS involvement, are more common in immunocompromised patients. Symptoms may vary and include headache, altered mental status, focal neurological signs, seizures, and signs of increased intracranial pressure.

#### 4.1.3. Diagnosis

Imaging plays a crucial role in diagnosing CNS infections, particularly since neurological symptoms can sometimes be overlooked, especially in patients with disseminated blastomycosis. MRI is the most sensitive technique, capable of identifying single or multiple ring-enhancing lesions without a predominant area of the brain involved, as well as meningeal enhancement in cases of meningitis [131].

Confirming CNS blastomycosis through laboratory methods can be challenging. The gold standard remains the growth of the fungus in microbiological cultures. However, a rapid presumptive diagnosis can often be obtained through histopathologic examination of affected tissues. In cases of meningitis, CSF analysis can provide valuable information, typically showing lymphocytic pleocytosis or, less commonly, neutrophilic predominance, elevated protein levels, and normal or decreased glucose levels [131]. CSF culture, however, has poor sensitivity [132]. For patients with mass lesions, a biopsy may be necessary for diagnosis. In some instances, the diagnosis of CNS blastomycosis can be presumed without biopsy in patients with confirmed non-CNS blastomycosis who exhibit abnormal neurological findings or imaging.

An enzyme immunoassay (EIA) that detects galactomannan in the cell wall of *B. dermatitidis* can also be useful in the rapid diagnosis of blastomycosis. It is usually performed on urine or serum but can also be useful with BAL fluid or CSF samples. Its sensitivity ranges from 76% to 90% in urine and 56% to 82% in serum. However, it is important to note that there is frequent cross-reactivity between this test and *Histoplasma capsulatum* EIA [125].

#### 4.1.4. Treatment

Blastomycosis with CNS involvement should be initially treated with liposomal amphotericin B (5 mg/kg per day) for four to six weeks, followed by an oral azole for a minimum of 12 months. Some patients, especially those who are immunosuppressed or experience relapsing CNS blastomycosis, may require lifelong therapy to prevent recurrence [133]. Possible options for azole therapy include itraconazole, voriconazole or fluconazole, although the lack of clinical trials comparing these drugs does not allow for a clear optimal choice of azole [133]. However, due to its excellent CSF penetration and increasing evidence of effectiveness in case reports of CNS blastomycosis [132], voriconazole (200 to 400 mg twice daily) has been increasingly suggested as the preferred option for oral step-down therapy, followed by itraconazole and fluconazole [125,130].

#### 4.1.5. Prognosis

CNS blastomycosis is usually associated with good outcomes when treated with the current standard-of-care antifungal therapy. Two studies reviewing cases of CNS blastomycosis due to *B. dermatidis* reported a mortality rate of approximately 18% in both series, although not all the fatalities were necessarily attributed to treatment failure or the infection itself [28,132]. The neurological outcomes of CNS infections caused by *B. helicus* or *B. percursus* remain uncertain due to a lack of sufficient evidence. Nevertheless, when patients adhere to treatment, favorable neurological outcomes are typically observed.

### 4.2. Sporothrix Species

#### 4.2.1. Definition and Epidemiology

*Sporothrix* species are dimorphic fungi, presenting as a mold in the environment and a yeast while in tissue [131]. Classified within the *Sporothrix* genus is a group of at least six phylogenetically closely related species, named the *S. schenckii complex*. Relevant species within this complex include *S. brasiliensis*, *Sporothrix schenckii sensu strictu,* and *Sporothrix globosa* [134]. It is mostly found in sphagnum moss, decaying wood, plants, and soil. Contamination usually occurs through accidental inoculation of organic material via animal scratches and bites, as well as through inhalation [131].

*Sporothrix* species are endemic in Mexico, Costa Rica, Guatemala, South America, South Africa, India, Japan, China, and Australia. In the southern and southeastern regions of Brazil, most cases are attributed to *S. brasiliensis*. Over the past decade, *S. brasiliensis* has caused significant outbreaks in Brazilian cities such as Rio de Janeiro and São Paulo, primarily due to zoonotic transmission from cats. Recent outbreaks have also been reported in Argentina and Panama [116]. In recent years, sporadic cases of sporotrichosis have been reported in Italy, Portugal, and Greece. These cases were mostly imported, although one case of sporotrichosis caused by *Sporothrix globosa* was reported as autochthonous in Portugal [135].

In a murine model, *S. brasiliensis* was identified as the most virulent member of the *Sporothrix schenckii complex*. This pathogen produces large amounts of urease and melanin, which serve as virulence factors facilitating tissue invasion and evasion from the immune system [135]. Other *Sporothrix* species typically cause cutaneous or lymphocutaneous infections. Due to its virulence and zoonotic transmission, *S. brasiliensis* infection has been associated with more severe clinical presentations.

In the last decade, a total of 36 cases of CNS sporotrichosis were described [46,47,48,49,50,51,52,53,54,55,56]. Among these, 30 cases occurred in patients with recognized risk factors: 26 with HIV infection, 2 with a history of alcoholism, 1 with chronic steroid use and 1 renal transplant recipient [46,47,48,49,50,51]. The predominant neurological manifestation was meningitis, occasionally accompanied by hydrocephalus. There was one case of intracranial abscesses. Additionally, five cases were identified in hosts with no known immunosuppression or risk factors. These included three cases of chronic meningitis by *S. brasiliensis* [52,53] and two cases involving S. *schenckii*—one with meningitis and one with intramedullary lesions [54,55]. The additional case had no description of immune status or species of *Sporothrix* isolated in the CSF [56].

#### 4.2.2. Clinical Features

*Sporothrix* species usually cause infections that are limited to the skin, subcutaneous tissue, and regional lymph nodes. Meningitis and other central nervous system infections are rare and mostly occur in immunosuppressed patients, such as those living with HIV, transplant recipients, cancer patients, individuals with alcoholism, diabetes mellitus, or those undergoing immunosuppressive therapies [46,136]. However, there has been an increasing number of disseminated cases in immunocompetent patients.

CNS sporotrichosis can manifest as an isolated chronic meningitis or secondary to widespread dissemination. Isolated CNS involvement is rare but typically occurs in immunocompetent patients. Most patients with CNS involvement also have concomitant cutaneous disease, with CNS invasion often occurring via hematogenous spread. In a study at a Brazilian reference center from 1999 to 2020, 53 patients with disseminated sporotrichosis underwent a lumbar puncture. Among them, 17 patients were diagnosed with meningitis (32%). All patients had concomitant cutaneous infection. Among the 17 meningitis cases, the most prevalent comorbidity was HIV infection (88%), followed by chronic steroid use (6%) and alcoholism (6%) [46].

Chronic meningitis usually presents with symptoms persisting over weeks to months such as headache, ataxia, and confusion. In severely immunosuppressed patients, such as those with HIV/AIDS and a CD4 T-cell count below 100 cells/mm^3^, meningitis symptoms can manifest more acutely. These symptoms are often a consequence of disseminated cutaneous sporotrichosis [131].

#### 4.2.3. Diagnosis

The CSF pattern and radiological findings in CNS sporotrichosis are typically similar to other causes of chronic meningitis. CSF findings often include elevated protein levels, decreased glucose levels and pleocytosis, predominantly mononuclear [131]. In endemic settings, tuberculous meningitis is the primary differential diagnosis. Additional differential diagnosis include other fungal infections, slow-growing bacterial infections (such as syphilis and brucellosis) and malignancies [52].

CSF culture is the gold standard for diagnosing CNS sporotrichosis, but it can take up to 4 weeks to obtain results. PCR or matrix-assisted laser desorption/ionization time-of-flight (MALDI-TOF) mass spectrometry-based methods have become indispensable tools for accurate species identification in many clinical laboratories [134]. The role of indirect enzyme-linked immunosorbent assay (ELISA) in CSF is not yet standardized, but it has demonstrated high sensitivity and specificity in diagnosing meningeal sporotrichosis [137].

Brain imaging typically reveals either normal findings or meningeal enhancement. In some cases, imaging may also show signs of vasculitis, infarcts, or hydrocephalus. Parenchymal lesions have been occasionally described in immunosuppressed patients.

Diagnosis is often presumptive, based on a positive culture or compatible histopathology from another involved site (usually the skin) in cases of disseminated disease, in a patient presenting with symptoms and/or signs of meningitis [131].

#### 4.2.4. Treatment

The recommended treatment regimen for CNS sporotrichosis typically involves initial therapy with liposomal amphotericin B (5 mg/kg daily) for 4-6 weeks, followed by oral itraconazole (200 mg twice daily) for a total of 12 months of therapy. Nonetheless, it is important to note that itraconazole penetrates poorly into the CNS. Although other azoles have been used in some cases, fluconazole and voriconazole have shown poor response rates and are not recommended for treating sporotrichosis. Posaconazole may be considered as an alternative for step-down oral therapy at a dose of 300 mg daily, especially when itraconazole is not tolerated, as it has some activity against *Sporothrix* species [131]. In certain situations, such as uncontrolled HIV infection, relapsing infection, or ongoing immunosuppressive treatments, lifelong maintenance therapy with azoles may be necessary.

#### 4.2.5. Prognosis

The clinical outcomes of chronic meningitis due to *Sporothrix* species in immunosuppressed patients are poor, with an overall mortality rate above 50% in some series [46]. In the previously mentioned cohort of 17 cases of *S. brasiliensis* meningitis, the mortality rate in immunosuppressed hosts was 64.7%. The same study reported a significantly lower mortality rate of 27.8% in patients with disseminated sporotrichosis without neurological involvement. Additionally, relapses were frequently observed [46].

### 4.3. Talaromyces marneffei

#### 4.3.1. Definition and Epidemiology

*Talaromyces marneffei*, formerly known as *Penicillium marneffei*, is a rare dimorphic fungus first isolated in 1956, existing as a yeast at 37 °C and a mold at 25 °C [57,138]. The first documented human infection case was reported by Disalvo in 1973, involving a 61-year-old American missionary with Hodgkin’s lymphoma who lived in Southeast Asia [139].

It is a geographically restricted opportunistic fungus capable of causing life-threatening infections in immunocompromised hosts, with rare occurrences in immunocompetent individuals [57]. This fungus is prevalent in Southeast Asia [57], ranking among HIV-infected individuals in this region as the third most common opportunistic infection [140]. However, it is rarely seen in non-endemic areas [57,138]. Sporadic cases have been reported among people from America, Europe, Korea, Singapore, and Africa who have either lived in or visited these endemic areas [57].

To date, bamboo rats are believed to be the sole reservoir of *T. marneffei* [66]. There is currently no conclusive evidence linking occupational exposure to *T. marneffei* infection. However, in a study conducted by Li et al., seven out of ten patients were farmers [66]. Infection rates exhibit notable seasonal variation, with higher incidence observed during the rainy season [141].

*Talaromyces marneffei* mainly affects immunocompromised individuals, particularly those with AIDS, especially when the CD4+ T cell counts are below 50 cells/μL. It is estimated that these patients account for 88% of those infected with this fungus, and approximately 50,000 HIV-positive patients are infected with *T. marneffei* annually in high-risk areas, with incidence expected to rise each year [66].

*T. marneffei* has shown a rising incidence among non-HIV infected individuals, driven particularly by increased incidence of malignancies, immunosuppressive therapies, and organ transplantation [142]. Infection in immunocompetent patients, including children, has been documented in rare instances [57,58]. In comparison to HIV-infected patients, those without HIV infection typically present as older individuals with higher white blood cell counts and higher mortality rates [141].

The thermally dimorphic fungus proliferates within macrophages, disseminates via the reticuloendothelial system and primarily targets the skin, lungs, liver, spleen, lymph nodes, and circulatory system [138]. Involvement of the CNS is extremely rare (<1%) and mainly presents as meningitis or meningoencephalitis [66].

A total of 21 cases *T. marneffei* CNS infections were identified [57,58,59,60,61,62,63,64,65,66,67,68], mainly presenting as meningitis, meningoencephalitis, or intracranial lesions: 12 cases were associated with AIDS [65,66,67], one occurred in a non-HIV immunosuppressed patient [68], and eight cases were observed in patients without known immune deficiencies [57,58,59,60,61,62,63,64].

#### 4.3.2. Clinical Features

*T. marneffei* infection is divided into two types: localized and disseminated, the latter being the prevailing form of presentation in people with advanced HIV infection. The localized form of the disease is usually characterized by skin and subcutaneous involvement, manifested primarily as localized umbilicated skin lesions, but reports of Sweet’s Syndrome are not uncommon [141]. Cutaneous lesions represent the most specific yet late expressions of talaromycosis, with central-necrotic papules appearing on the face, trunk, and extremities in 40% to 70% of patients [143].

Clinical manifestations of disseminated infection encompass symptoms like fever, shivering, emaciation, anemia, hepatosplenomegaly, lymphadenopathy, abdominal pain or diarrhea, pulmonary involvement with cough and expectoration, umbilicated skin nodules, and the formation of abscesses [144]. When CNS involvement is observed, it is typically in the form of meningitis or meningoencephalitis, with concomitant occasional intracranial infectious lesions also possible. Isolated CNS involvement has also been previously reported [66].

#### 4.3.3. Diagnosis

In HIV-infected patients with CD4 count <100 cells/mm^3^ who present with a subacute febrile syndrome, including changes in mental status, and who live or have traveled to endemic regions, disease due to *T. marneffei* should be considered.

CSF profile of AIDS-associated *T. marneffei* CNS infection is similar to those of other types of fungal meningitis, including cryptococcal meningitis. Definitive diagnosis is made after isolation in CSF cultures. On a retrospective study of ten patients in China who were affected with AIDS-associated CNS infection by *T. marneffei*, all patients had positive CSF cultures with a median duration of growth of 11.5 days and 30% of patients had identification of *T. marneffei* in other non-CSF [66]. However, it is of note that either longer (up to 28 days) and shorter periods of incubation until fungal growth (specifically 3 days) have also been reported in the literature [140,141].

Presumptive diagnosis of CNS talaromycosis is made when positive cultures or histopathology are obtained from blood or another affected site in a patient with disseminated talaromycosis, along with accompanying symptoms and signs of meningitis [131]. Antigen detection and molecular diagnostics for talaromycosis on PCR amplification and sequence identification of specific regions are promising rapid diagnostics currently being evaluated [138,143].

The neuroimaging exams in *T. marneffei* CNS infected patients usually exhibit abnormal findings, mainly manifested as ventricular dilatation and intracranial lesions. However, ventriculomegaly is also commonly observed in the neuroimaging of individuals with advanced HIV/AIDS, attributed to HIV encephalopathy leading to cerebral atrophy, meaning that the direct effect of *T. marneffei* in the pathophysiology of ventricular dilatation remains unclear [66]. 

#### 4.3.4. Treatment

Amphotericin B formulations are the most common and successful antifungal drugs for CNS fungal infections. This agent has fungicidal activity and is superior in efficacy to fluconazole, itraconazole, and voriconazole against *T. marneffei*, although these antifungal agents are also effective [66]. There are at least two reported cases where the CNS infection was successfully treated with voriconazole [57,138].

Currently, the recommended first-line treatment for *T. marneffei* CNS infection includes induction therapy with liposomal amphotericin B (3–5 mg/Kg/day) for 2 weeks, followed by consolidation therapy with oral itraconazole (200 mg twice daily) for 10 weeks [143].

Extension of the initial course of amphotericin B may be required until cerebrospinal fluid fungus cultures become negative. Intrathecal administration is not recommended for patients with CNS involvement as intravenous administration is often effective [58].

After this period, immunocompromised host should transition to maintenance therapy with oral itraconazole (200 mg/day) [66]. Maintenance treatment in HIV-patients should be continued with itraconazole until CD4+ count >100 cells/mm^3^ and virologic suppression for ≥6 months in response to antiretroviral therapy (ART) [143]. In non-HIV patients, the precise duration of effective maintenance therapy is not well-established. It seems reasonable to consider voriconazole over itraconazole due to its better CNS penetration, but there are no clinical reports to support this.

To improve outcomes, ART may be initiated as early as one week after starting amphotericin B induction therapy for talaromycosis treatment [66,143].

#### 4.3.5. Prognosis

Given the long culture incubation time and high disease mortality, heightened clinical suspicion and prompt empirical treatment with a CNS-penetrating antifungal drug, such as amphotericin B, are critical in the management of these patients. In CNS infection, mortality is estimated at 80% [143]. Low CD4+ T cell counts may be associated with higher mortality [66].

## 5. Molds

### 5.1. Scedosporium Species

#### 5.1.1. Definition and Epidemiology

*Scedosporium* species are saprophytic fungi with a worldwide distribution. This species is commonly found in human-impacted environments such as polluted water and sewage, agricultural soils, gardens, urban parks, or hydrocarbon-contaminated soils [145,146]. The genus *Scedosporium* comprises various species, including *S. aurantiacum*, *S. minutisporum*, *S. desertorum*, *S. cereisporum*, *S. dehoogii*, and *S. apiospermum*. *S. apiospermum* is now recognized as a complex that includes, among others, the most frequently isolated species of *S. apiospermum* and *S. boydii* [70].

Immunossupression is the main risk factor for invasive *Scedosporium* species infections [145,147]. However, these infections can also occur in immunocompetent hosts, typically following traumatic injuries. Notably, *S. apiospermum* complex CNS infections have been closely linked to drowning events in immunocompetent hosts, such as those resulting from tsunamis, storms, and earthquakes [88,89,146].

*Scedosporium* species has been increasingly identified in clinical samples and is reported as the second most common cause of non-*Aspergillus* mold infections after mucormycosis [148]. This species is intrinsically resistant to many current antifungal agents, contributing to a high mortality rate and growing concern about these emergent fungal pathogens [149].

In the 42 collected cases of *Scedosporium* species CNS infections [69,70,71,72,73,74,75,76,77,78,79,80,81,82,83,84,85,86,87,88,89,90,91,92,93,94,95,96,97,98,99], 19 occurred in immunocompromised patients [69,70,71,72,73,74,75,76,77,78,79,80,81,82,83,84,85]. Interestingly, among the immunocompromised patients, two solid organ transplant recipients developed CNS infections after receiving solid organ transplants from donors who died from drowning [75,76]. Eighteen cases involved immunocompetent patients, all associated with trauma or near-drowning events [86,87,88,89,90,91,92,93,94]. The remaining five cases occurred in patients over 65 years-old, with no other known cause of immune deficiency [95,96,97,98,99]. Among these, four patients had no history of traumatic or near-drowning events, suggesting that immunosenescence alone may be a risk factor for invasive scedosporiosis. Neurological manifestations primarily included abscesses, but also encompassed ventriculitis, hydrocephalus, meningitis, septic emboli and mycotic aneurysms.

#### 5.1.2. Clinical Features

*Scedosporium* species can cause localized infections such as cutaneous, eumycetoma, ocular, muscle, joint, and bone infections. These infections can affect any organ, with a predilection for the skin (28%), lungs (25%), central nervous system (24%), and eyes (23%) [150]. *S. apiospermum* complex is a frequent airway colonizer and is the second most frequently isolated filamentous fungus from the airways of cystic fibrosis patients [151]. Additionally, it may trigger inflammatory responses similar to those observed in allergic bronchopulmonary aspergillosis.

Disseminated infections generally occur in immunocompromised patients, with CNS involvement representing a severe manifestation. In such cases, the disease often progresses more rapidly than the diagnostic process [70]. CNS infections result from hematogenous dissemination from other foci, following trauma or iatrogenic procedures, or via contiguous spread from the sinuses. In immunocompetent hosts, *Scedosporium* species CNS infections are mostly associated with traumatic inoculation after near-drowning episodes [146]. After near-drowning events, symptoms may manifest weeks after the incident. Onset of neurological symptoms in these patients should prompt suspicion of CNS infection, even in the absence of pneumonia [87].

The primary clinical presentation of CNS infection includes single or multiple brain abscesses, although it can also lead to meningitis and ventriculitis. Abscesses may occur in the cerebral hemispheres, brainstem, cerebellum, and spinal cord, without any anatomical preference.

#### 5.1.3. Diagnosis

There are no pathognomonic clinical symptoms or radiological findings of *Scedosporium* infection. Definite diagnosis is based on isolation via culture, with direct microscopy and histopathology also being helpful [146]. However, distinguishing *Scedosporium* species from *Aspergillus* species using microscopic and histopathological methods is challenging [73].

Molecular-based identification methods, such as DNA sequence-based analysis, are the current gold standard for fungal identification. MALDI-TOF mass spectrometry has become increasingly available, as it is a relatively efficient and fast diagnostic method, but the available commercial databases are not always adequate for a consistent identification of all pathogenic *Scedosporium* species [149]. Fungal cell wall components, such as (1,3)-beta-d-glucan, may be helpful in suggesting fungal etiology of infection, although not specific.

#### 5.1.4. Treatment

Treatment of *Scedosporium* species infections is challenging due to the intrinsic resistance of these fungi to many currently available antifungal agents. Due to species-specific susceptibility patterns, accurate identification of the causative species is crucial. Furthermore, the MICs for certain antifungal drugs can vary between isolates within each species, highlighting the importance of determining the MICs for all clinically significant isolates.

All *Scedosporium* species are resistant in vitro to amphotericin B, flucytosine and fluconazole, and demonstrate reduced susceptibility to echinocandins. Voriconazole and posaconazole typically exhibit the lowest MICs against *Scedosporium* species [150]. According to European guidelines, voriconazole monotherapy is the recommended first-line treatment for *Scedosporium* species infections [152,153].

Treatment should include adjuvant surgery for the removal of infected tissues and reversal of immunosuppression when feasible. The optimal duration of treatment is unknown, but most surviving cases reported prolonged treatment (more than 1 year), often combined with surgical drainage when possible.

#### 5.1.5. Prognosis

Given the nonspecific symptoms, laboratory results, imaging and CSF findings, the diagnosis and appropriate treatment of these CNS infections are frequently delayed. Mortality rates for patients with CNS scedosporiosis vary significantly, ranging from approximately 30% in those treated with surgical drainage plus medical therapy to 74% in those treated with antifungals alone [154]. The intrinsic resistance to the major classes of antifungal contributes to this outcome. Unsurprisingly, solid organ transplant recipients, CNS infections and disseminated infections are associated with higher mortality rates in scedosporiosis [150].

### 5.2. Lomentospora prolificans

#### 5.2.1. Definition and Epidemiology

*Lomentospora prolificans*, formerly known as *Scedosporium prolificans*, was renamed due to its phylogenetic distance from the *Scedosporium* genus [155]. This species is a saprophytic filamentous fungus usually isolated from soil and predominantly found in Australia, European regions (particularly Spain), and Southern USA [145].

*L. prolificans* primarily affects immunocompromised patients, but can also rarely infect healthy individuals. Immunosuppressed patients, particularly those with hematological malignancies, hematopoietic stem cell, or solid organ transplantation, are more susceptible to disseminated or invasive disease [145,147]. In contrast, these infections are rare in people living with HIV or primary immunodeficiencies [145].

This species has increasingly been identified as an important cause of non-*Aspergillus* mold infections. A retrospective study from California, USA, reported that *L. prolificans* infections accounted for 35% of all non-*Aspergillus* invasive mold infections [156]. In a multicenter study in Australia, which compiled 165 episodes of non-*Aspergillus* mold infections, *Scedosporium*/*Lomentospora* species were responsible for 33.3% of the infections, with *Lomentospora prolificans* accounting for approximately 50% of these infections [148]. The major comorbidities included hematologic malignancy in 45%, diabetes in 23%, stem cell or solid organ transplant in 19% and 14%, respectively, and chronic lung disease in 16%. 15% of the patients had no identifiable comorbidities. Of the 19 total cases of CNS infection, 6 (32%) were caused by *Scedosporium/Lomentospora* species. However, the publication did not specify how many of these were due to *Scedosporium* species or *L. prolificans*.

*L. prolificans* is inherently resistant to all currently available antifungal agents, rendering infections exceptionally challenging to manage [149], with increased mortality in cases involving the central nervous system [83].

A total of 22 cases of CNS lomentosporiosis were identified, 20 of which occurred in immunocompromised patients [83,86,87,100,101,102,103,104,105,106,107,108,109]. Neurological involvement included meningitis, abscesses, cerebral emboli, and mycotic cerebral aneurysms. The sole case described in an immunocompetent patient presented, after a near-drowning episode, with pneumonia and multiple brain and cervical cord lesions [87]. The result of this patient´s CSF cultures was negative, but sputum cultures were positive for both *Scedosporium apiospermum* and *Lomentospora prolificans*, leaving it unclear which pathogen, or if both, were responsible for the CNS involvement. Another literature review described one case of CNS *L. prolificans* infection in a child after direct inoculation, but the child’s immune status was not mentioned [86].

#### 5.2.2. Clinical Features

Infection typically occurs through inhalation of airborne conidia or traumatic inoculation from contaminated environmental sources. Clinical presentations can range from colonization to localized or disseminated infection [157]. Distinguishing between colonization and infection can be challenging. *L. prolificans* frequently colonizes the airways of patients with structural changes, such as those with in cystic fibrosis and lung transplant patients [145].

*L. prolificans* infection can affect the skin, soft tissue, muscle, bone and joint, eye, lungs, heart, and CNS. The most common presentation is disseminated infection [157]. *L. prolificans* is prone to dissemination as it produces conidia in body fluids and tissues, allowing it to spread through the bloodstream [153]. Severe immunosuppression, including neutropenia, is the highest most significant risk factor for disseminated disease [158].

This fungus is also characterized by its neurotropism, which, combined with an ineffective response of phagocytes in the CNS against *L. prolificans*, explains its propensity to invade and cause CNS infection [157]. Common neurological presentations include meningitis, abscesses, or complications of hematological dissemination such as emboli or mycotic aneurysms.

#### 5.2.3. Diagnosis

Direct microscopy is valuable for diagnosing invasive lomentosporiosis. However, various hyaline molds exhibit similar morphologies in tissue samples. Thus, culture remains the gold standard for diagnosis, with the added benefit of allowing susceptibility testing in a setting of expected resistance to multiple antifungals.

In contrast to most mold infections, fungemia by *L. prolificans* is frequent, positive blood cultures in approximately 75% of the patients [158]. *L. prolificans* grows on standard mycological media, like Sabouraud’s dextrose agar. However, blood cultures typically yield positive results late in the course of the disease, limiting their diagnostic usefulness. MALDI-TOF mass spectrometry is increasingly utilized and a promising rapid and accurate method for identifying *L. prolificans*. Molecular diagnostic techniques, such as PCR or DNA sequencing, are also becoming more readily available and can help in earlier identification of these invasive infections, albeit as adjuncts to conventional laboratory testing [157].

The clinical utility of serum 1, 3-beta-D-glucan is controversial, with conflicting results in *L. prolificans* infection [158].

#### 5.2.4. Treatment

The management of *L. prolificans* infections poses a significant challenge due to its intrinsic resistance to the majority of available antifungal therapies. *L. prolificans* is a pan-antifungal resistant species, with voriconazole showing the highest in vitro activity. Notably, *L. prolificans* demonstrates resistance in vitro to amphotericin B, flucytosine, fluconazole, itraconazole and echinocandins. In vitro studies have demonstrated synergy of terbinafine with voriconazole, and to a lesser extent with posaconazole, suggesting potential therapeutic benefits in the treatment of *L. prolificans* infections [149].

Combination antifungal therapy has shown higher survival rates compared to monotherapy [156]. Thus, the European Confederation of Medical Mycology guidelines recommend the use of voriconazole-based combination antifungal therapy for *L. prolificans* infections, particularly voriconazole combined with terbinafine and an eventual third antifungal agent [152]. Duration of treatment is not well established, although treatment should generally be continued until all signs and symptoms of infection have resolved. Most cases of survival were patients submitted to prolonged treatment of more than 1 year, combined with surgical drainage when possible.

The intrinsic resistance to current treatments highlights the need for new antifungal agents. Olorofim, a novel antifungal with promising results and low in vitro MICs in *L. prolificans*, is currently in Phase IIB clinical trials for the treatment of invasive mold infections, including by *L. prolificans*, in patients with few treatment options [158].

#### 5.2.5. Prognosis

Lomentosporiasis is an infection with very high mortality rates of around 87–95%, on account of the propensity of highly immunocompromised patients to acquire these infections, the intrinsic resistance to major classes of antifungal therapies of this species, and its neurotropism [153,158].

## 6. Discussion

Fungal infections of the central nervous system, once considered rare, are becoming more prevalent. Over the past decade, several fungi have emerged as significant pathogens capable of causing life-threatening CNS infections. This article reviews the broad clinical characteristics and the documented cases in the last decade of seven unconventional CNS fungal pathogens. While the exact burden of these diseases remains difficult to ascertain, each fungal species has distinct factors contributing to its increased incidence in CNS infections. *Candida auris* has emerged globally as a multidrug-resistant pathogen causing healthcare-associated outbreaks. *Trichosporon* spp. has been increasingly diagnosed as a cause of infection in both immunosuppressed and immunocompetent patients. *Blastomyces* spp. has shown an expansion of its geographical endemic areas, as well as discovery of novel species. *Sporothrix* spp. has been associated with large outbreaks due to zoonotic transmission. *Talaromyces marneffei* has also been reported as a cause of CNS infections in immunocompetent or non-HIV infected hosts, in addition to its well-known predilection for people living with HIV. *Lomentospora prolificans* is increasingly recognized as a devastating cause of CNS infections in immunosuppressed hosts, with pan-resistance to all current antifungal agents. *Scedosporium* spp., besides being an important cause of disease in immunosuppressed patients, is also frequently associated with CNS infections after near-drowning events linked to natural disasters.

Diagnosing CNS fungal infections presents significant challenges. The nonspecific clinical manifestations often mimic other neurological disorders, leading to potential misdiagnosis or delayed treatment. Imaging findings are equally nonspecific. Contributing to these challenges is the lack of rapid and reliable diagnostic methods. Traditional methods like culture and histopathology, while useful, are often slow to provide answers and can be hampered by the concomitant presence of bacterial infections. Therefore, more advanced diagnostic techniques such as molecular methods, mass spectrometry, or serological tests are increasingly necessary to achieve accurate and timely diagnosis.

Treatment of CNS fungal infections is fraught with challenges. The inherent resistance of certain fungi to available antifungal agents and their limited penetration into the CNS can significantly hinder treatment choices and efficacy. The need for prolonged courses of antifungal therapy, sometimes lasting months to years, further complicates management and increases the risk of adverse drug effects and interactions.

These challenges contribute to the high mortality rates associated with CNS fungal infections. The combination of diagnostic difficulties, treatment limitations, increased vulnerability of immunosuppressed patients, and changing environmental factors underscores the urgent need for improved diagnostic tools, more effective antifungal agents with better CNS penetration, and comprehensive management strategies. Addressing these needs is crucial for reducing the burden of these life-threatening infections and improving outcomes for affected patients.

A limitation of this study is the lack of published information, as most of the existing literature on these rare fungal infections of the CNS consists of case reports. This scarcity makes it difficult to identify evidence-based guidance for the optimal approach to these infections. Consequently, clinical decisions are often reliant on expert opinion. This review aims to provide valuable insight to assist in decision-making in such challenging situations.

## Figures and Tables

**Table 1:** Clinical characteristics.

Fungus	Morphology	Geographic Distribution	Host Immune Status	Main Presentation
*Candida auris*	Yeast	Worldwide (sparing only Antartica)	Immunocompromised. Other risk factors: prolonged healthcare contact, indwelling devices, use of broad-spectrum antibiotics or antifungal agents, skin colonization	Asymptomatic colonization to invasive disease. Candidemia is frequent. Other manifestations: urinary tract infections, otitis, wound infections, skin abscesses, osteomyelitis, myocarditis.
*Trichosporon* species	Yeast	Worldwide	Superficial infections: both immunocompetent and immunocompromised. Invasive infections: mainly immunocompromised. Other risk factors: poor hygiene, broad-spectrum antibiotics, burns, invasive devices	Superficial infections: White piedra, onycomycosis and cutaneous lesions.
*Blastomyces* species	Dimorphic fungi	*B. dermatidis*: parts of USA and Canada *B. percursus*: Middle East, Africa *B. helicus*: Western USA and Canada *B. gilchristii*: Wisconsin, Ontario *B. emzantsi*: South Africa	Both immunocompetent and immunocompromised *B. helicus:* Immunocompromised	Predominantly pulmonary disease. *B. percursus*: Higher prevalence of cutaneous and bone manifestations, as well as pulmonary disease.
*Sporothrix* species	Dimorphic fungi	Worldwide (endemic in Latin America, South Africa, India, Japan, China, Australia)	Cutaneous: usually immunocompetent. CNS: usually immunocompromised (including alcoholism, Diabetes mellitus).	Limited cutaneous to disseminated disease
*Talaromyces marneffei*	Dimorphic fungi	Southeast Asia (Thailand, Southern China, Northeastern India and Vietnam)	Predominantly in immunocompromised, rarely in immunocompetent hosts.	Localized, mainly as cutaneous. Dissemination is more common in immunocompromised: lungs, liver, skin, lymph nodes, and CNS
*Scedosporium* species	Mold	Worldwide	Immunocompromised and immunocompetent. Other risk factors: trauma or near-drowning events, chronic lung disease, elderly	Asymptomatic pulmonary colonization, bronchopulmonary mycosis, traumatic cutaneous infections, eumycetoma.
*Lomentospora prolificans*	Mold	Australia, European regions (particularly Spain), Southern USA	Immunocompromised. Other risk factors: chronic lung disease, Diabetes mellitus	Asymptomatic colonization to invasive disease. Disseminated disease is the most common.

CNS: Central nervous system; USA: United States of America.

**Table 2:** Literature review of CNS infections.

Fungus	Number of Cases (Type of CNS Involvement)	CNS Involvement	CNS Imaging	Antifungal Susceptibility Testing	Recommended Treatment	Reported Attributed Mortality (Rate)	References
*Candida auris*	10	Neurosurgical device infections, meningitis, and ventriculitis	Nonspecific	R 90% to fluconazole, 30% to amp-B, 5% to echinocandins	Guided by susceptibility testing (echinocandins not recommended)	2/7 (29%)	[5,6,7,8,9,10,11,12]
*Trichosporon* species	5	Meningoencephalitis, hydrocephalus, brain abscesses and intraventricular fungal ball	Hydrocephalus, circular ring-enhancing brain lesions, abnormal meningeal enhancement, or plaque-like enhancing medullar lesions	Possible high-MICs to amp-B, R echinocandins	Voriconazole	2/5 (40%)	[13,14,15,16,17]
*Blastomyces* species	82	Brain abscesses, meningitis, ventriculitis, hydrocephalus	Multiple ring-enhancing lesions with no predominant area of the brain involved, enhance of meninges	R echinocandins	Lamp-B followed by an oral azole	8/73 (11%)	[18,19,20,21,22,23,24,25,26,27,28,29,30,31,32,33,34,35,36,37,38,39,40,41,42,43,44,45]
*Sporothrix* species	36	Subacute and chronic meningitis	Nonspecific	R echinocandins, poor response to voriconazole and fluconazole	Lamp-B followed by oral itraconazole	23/35 total (66%) 22/30 immunosuppressed (73%) 1/5 immunocompetent (20%)	[46,47,48,49,50,51,52,53,54,55,56]

Amp-B: Amphotericin B; Lamp-B: liposomal amphotericin B; MIC: minimal inhibitory concentrations R: resistant

**Table 3:** Literature review of CNS infections.

Fungus	Number of Cases (Type of CNS Involvement)	CNS Involvement	CNS Imaging	Antifungal Susceptibility Testing	Recommended Treatment	Reported Attributed Mortality (Rate)	References
*Talaromyces marneffei*	21	Meningitis or meningoencephalitis rarely space occupying lesions	Nonspecific	R echinocandins, fluconazole, posaconazole, isavuconazole	Lamp-B followed by oral itraconazole	2/19 (20%)	[57,58,59,60,61,62,63,64,65,66,67,68]
*Scedosporium* species	42	Predominantly abscesses, but also ventriculitis, hydrocephalus, meningitis, septic emboli and myco(tic aneurysms	Space occupying lesions, ventriculomegaly, ventriculitis, septic emboli, mycotic aneurysms	R echinocandins, amp-B, fluconanzole, itraconazole	Voriconazole	18/36 total (50%) 9/17 immunosuppressed (53%) 4/5 elderly (80%) 5/14 immunocompetent (36%)	[69,70,71,72,73,74,75,76,77,78,79,80,81,82,83,84,85,86,87,88,89,90,91,92,93,94,95,96,97,98,99]
*Lomentospora prolificans*	22	Meningitis, abscesses, cerebral emboli, and mycotic cerebral aneurysms	Space occupying lesions, mycotic aneurysms	R echinocandins, amp-B, azoles, flucytosine, terbinafine	Voriconazole combined with terbinafine and an eventual third antifungal agent	8/10 (80%)	[83,86,87,100,101,102,103,104,105,106,107,108,109]

Amp-B: Amphotericin B; Lamp-B: liposomal amphotericin B; R: resistant.

## Data Availability

The original data presented in the study are openly available in PubMed at https://pubmed.ncbi.nlm.nih.gov, accessed on 3 October 2024.
